# Consultations—A Query

**Published:** 1877-06-20

**Authors:** 


### CONSULTATIONS—A QUERY.

Dr. J. J. G., writing from Providence, Miss., after agreeing with the views expressed in our last issue, in regard to consultations, asks: "What is a physician to do when he believes, or knows, the diagnosis and treatment in the case has been wrong, and he is called upon by intelligent friends of the patient to give them his honest opinion as to the nature of the case, and the treatment previously used—you thus find yourself in a *corner*—how are you to get out—by telling your honest opinion, or *lie* out to save a brother M. D.'s character?"

This would seem to be a poser at first sight; but, when it is considered that consultations are not called to sit in council, on the character, ability, or conduct of the attending physician, or to determine what ought to *have been done*, but, rather, to act upon the case as it stands, it is manifest that questions from the friends, or outside parties, in regard to the previous conduct of the case, are improper, and irrelevent to the objects in view, and ought not to be asked, and *should not be answered*, unless favorable to the attending physician. The ethics require that the directions agreed upon in consultation should be communicated by the physician first in attendance. No statement, or discussion of it, should take place before the patient, or his friends, except both parties are present, and consent thereto; "*and no opinions, or prognostications*, should be delivered which are not the result of previous deliberation and concurrence. * * * Neither by word nor manner should any of the parties to a consultation *assert, or insinuate*, that any part of the treatment pursued did not receive his assent."

In the event of irreconcilable difference of opinion, the fact should be kindly and respectfully stated to the friends, and "a third physician, if practicable, be called, to act as umpire. If circumstances prevent the adoption of this course, it must be left to the patient to select the physician in whom he is most willing to confide."

